# Comprehensive Modelling of the Hysteresis Loops and Strain–Energy Density for Low-Cycle Fatigue-Life Predictions of the AZ31 Magnesium Alloy

**DOI:** 10.3390/ma12223692

**Published:** 2019-11-08

**Authors:** Jernej Klemenc, Domen Šeruga, Aleš Nagode, Marko Nagode

**Affiliations:** 1 Faculty of Mechanical Engineering, University of Ljubljana, Aškerčeva 6, SI-1000 Ljubljana, Slovenia; jernej.klemenc@fs.uni-lj.si (J.K.); marko.nagode@fs.uni-lj.si (M.N.); 2 Faculty of Natural Sciences and Engineering, University of Ljubljana, Aškerčeva 12, SI-1000 Ljubljana, Slovenia; ales.nagode@ntf.uni-lj.si

**Keywords:** Magnesium AZ31, variable loading history, low-cycle fatigue, hysteresis-loop model, energy approach

## Abstract

Magnesium is one of the lightest metals for structural components. It has been used for producing various lightweight cast components, but the application of magnesium sheet plates is less widespread. There are two reasons for this: (i) its poor formability at ambient temperatures; and (ii) insufficient data on its durability, especially for dynamic loading. In this article, an innovative approach to predicting the fatigue life of the AZ31 magnesium alloy is presented. It is based on an energy approach that links the strain–energy density with the fatigue life. The core of the presented methodology is a comprehensive new model for tensile and compressive loading paths, which makes it possible to calculate the strain–energy density of closed hysteresis loops. The model is universal for arbitrary strain amplitudes. The material parameters are determined from several low-cycle fatigue tests. The presented approach was validated with examples of variable strain histories.

## 1. Introduction

To achieve a lightweight design that will fulfil the ever-growing ecological requirements and reduce fuel consumption, especially in the automotive and aerospace industries, new materials are being sought that demonstrate a good strength-to-weight ratio [1,2,3,4,5,6]. Magnesium alloys appear as a promising solution for these requirements as magnesium is one of the lightest metals, approximately 1.6 times lighter than aluminium alloys and about 4.5 times lighter than steel, while it has high strength, good machinability and good recyclability [4,5,7,8,9]. Various magnesium alloys exist. They are mostly suitable for casting purposes, e.g., AZ63 or AZ91 [10], although magnesium alloys such as AZ31 are available as sheet plates of several thicknesses that are suitable for forging purposes [4,8]. The use of the latter as the casting is not as widespread, mostly for two reasons. First, as AZ31 is poorly formable at ambient temperatures, it always requires forming at raised temperatures, typically over 230 ${}^{\circ }$C, which increases the production costs. Second, there is a lack of experimental data on dynamic loading, which makes the durability predictions of structural components manufactured from AZ31 either very challenging or not reliable [5,6,10].

Three deformation mechanisms interchange during the dynamic loading of AZ31, i.e., sliding, twinning and untwining [6], although twinning and untwining could be interpreted as a single mechanism. Sliding is also the dominant deformation mechanism in conventional metallic structural components such as steel and aluminium alloys, whereas twinning and untwining are more typical of magnesium and titanium alloys [4,6,11,12,13,14]. They are a consequence of the hexagonal close-packed crystal structure, where the twinning mechanism requires lower activation energy than pyramidal and prismatic sliding [4,6,13,14,15]. Therefore, the crystal structure will include an amount of twinned crystals before or with the activation of the sliding mechanism, especially in the compressive direction [10]. If the crystal structure has already been twinned under compressive loading and then the load is inverted into the tensile direction, the untwining mechanism occurs prior to the sliding mechanism in tension as the activation energy for the untwining is lower than the activation energy for the sliding. In contrast, sliding requires the smallest activation energy in the body-centred and face-centred cubic crystal structures typical for steel and aluminium alloys, regardless of the loading direction [6]. On the macroscopic scale, a symmetrical stress–strain response is obtained for the body-centred and the face-centred cubic crystal structures if the material is loaded with an alternating strain [16]. The interchange of the three mechanisms in the hexagonal close-packed crystal structure causes an asymmetrical shape of the stress–strain curve, typically also observed for AZ31 [6]. If such a response is to be simulated, the simulation model has to enable distinguishing between the mechanisms which occur either during the initial loading or during the tensile/compressive loading paths of the hysteresis loops.

The dynamic loading of metallic structures can lead to fatigue failure. Under severe loads, the material will fail due to low-cycle fatigue. The strain–life relation is usually used to describe the behaviour of the materials for the low-cycle fatigue. The strain–life relation provides the influence of the strain amplitude to the fatigue life of the material for the given dynamic ratio (e.g., typically R = −1). In addition to the strain amplitude, the stress amplitude and the mean stress drastically influence the fatigue life of the material, too. The prediction models for low-cycle fatigue life typically assume that the strain is the known variable, whereas the stress response has to be modelled. Especially for variable load histories, neglecting the influence of the stress amplitude or the mean stress, erroneous fatigue-life predictions might be observed. Damage parameters have therefore been introduced which consider the influence of the strain amplitude, the stress amplitude and the mean stress (e.g., Smith–Watson–Topper and Bergmann damage parameters [17]). Damage parameters however are based on the dissipated energy within a hysteresis loop and are therefore directly connected to the strain–energy density calculation. By obtaining the hysteresis loops in a given variable loading history, it is possible to calculate the damage contribution of every hysteresis loop to the total accumulated damage. Due to the rather complicated shape of the closed hysteresis loops for the AZ31 magnesium alloy, we present in this paper how an energy approach to calculating the fatigue life can be applied. In the literature, different energy approaches were applied to calculate the fatigue damage. They differ according to the definition of the critical damage. Some approaches link the fatigue-life damage to the static tensile curve (e.g., see Letcher et al. [18] and Ozaltun et al. [19]). Another approach is to link the energy to the Coffin–Manson durability curve (e.g., see Park and Nelson [20], Kabir and Yeo [21], and Jahed et al. [22]). We decided to follow a different approach in which the fatigue life is calculated according to the energy curve of the material, because this approach was already applied in the past to calculate the fatigue life for both the constant and variable strain amplitude loading of magnesium alloys (e.g., see Jahed et al. [23], Park et al. [24], Wang et al. [25] or Dallmeier et al. [26,27]).

**Figure 1:**
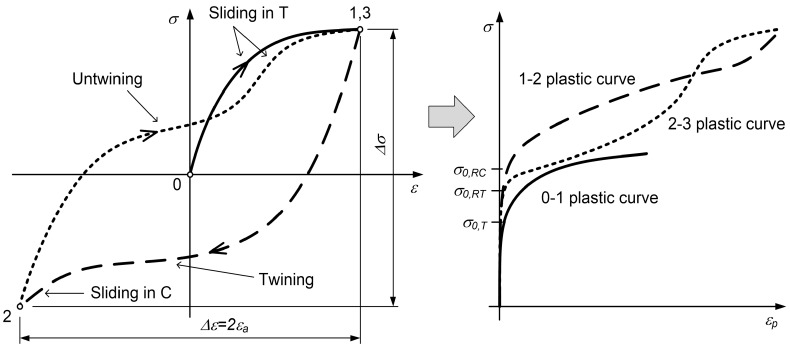
Initial tension loading and the closed hysteresis loop for the AZ31 magnesium alloy.

As we focused on the fatigue-life prediction under a variable loading history, it is important to be able to properly calculate the energy density of a hysteresis loop considering its position in the stress–strain space. Sigmoidal functions are used to describe the S-shaped stress–strain response of magnesium alloys [27,28,29,30]. Some researchers have decomposed the closed hysteresis loop of magnesium alloys into its elastic and plastic parts (e.g., Lee et al. [28] and Muhammad et al. [29]). They put a special emphasis on the modelling of the plastic part of the hysteresis loop. Since their experimental data ranged up to a strain amplitude of 6%, they observed the twinning and sliding plasticity mechanisms in both the tensile and the compressive loading paths. Even though the models of Lee et al. [28] and Muhammad et al. [29] are somewhat different, their basic idea is to have three different models for the hardening law(s). Their models can be summarised as follows (see also Figure 1):

1. A monotonic tensile curve (0–1) is a sum of the constant monotonic tensile yield stress $\sigma_{0,T}$ and the exponential curve with the generic form $A\cdot \left[1-\exp\left(-B\cdot \varepsilon_{p}\right)\right]$.
2. A compressive reverse loading path (1–2) is a combination of the constant monotonic compressive yield stress $\sigma_{0,\mathrm{RC}}$, the exponential curve $A\cdot \left[1-\exp\left(-B\cdot \varepsilon_{p}\right)\right]$ for modelling the twinning process and the sigmoidal function for modelling the sliding plasticity during the compression.
3. A tensile reverse loading path (2–3) is a combination of the constant monotonic tensile yield stress $\sigma_{0,\mathrm{RT}}$, the exponential curve $A\cdot \left[1-\exp\left(-B\cdot \varepsilon_{p}\right)\right]$ for modelling the untwining process and the sigmoidal function for modelling the sliding plasticity during the compression. The parameters for this model are different from the parameters of the second model.

For the modelling of the closed hysteresis loop, only the last two models are important, because our objective is to calculate the fatigue-life damage due to cyclic loading. The main problem linked to the models of Lee et al. [28] and Muhammad et al. [29] is that the elastic and plastic parts of the hysteresis loop are separated, since the Masing rule cannot be applied for the magnesium alloy. Furthermore, we noticed that the initial plastic part of the tensile and the compressive loading path is not well modelled with the sum of the initial yield strength and the exponential function:

(1) $$\sigma_{p}\;=\;\sigma_{0}\;+\;A\cdot \left[1\;-\;\exp\left(-\;B\cdot \varepsilon_{p}\right)\right].$$

It turned out that the plastic part of the Ramberg–Osgood equation [31] fits the experimental data much better. In addition, it can be applied to the elastic–plastic material curve relatively easily, which is well suited to our problem. Consequently, we used the full Ramberg–Osgood equation in the following form (see Dowling [32]) as a base function for the compressive and tensile loading paths:

(2) $$\varepsilon \;=\;\frac{\sigma }{E}\;+\;K\cdot {\left(\frac{\sigma }{E}\right)}^{n}\;⟶\;\varepsilon \;=\;RO\left(\sigma \right),$$

where *E* is the material’s Young’s modulus, *K* is a function of the yield strength and *n* is a hardening exponent. By adding a sigmoidal function, the transition from exhaustion of the twinning into to the sliding plastification mode during the tensile loading path can be described, as also noted by Dallmeier et al. [27]. However, the main advantage of the new model is its comprehensiveness. Namely, the cyclic behaviour of AZ31 can be correctly described parametrically and phenomenologically, regardless of the size and the position of the hysteresis loop in the stress–strain space, i.e., a single function with a single set of material parameters, without the need for interpolation between the hysteresis loops of different sizes, is required for the stress–strain modelling. The energy-density calculation is then possible using this new model for the parametric description of the compressive and tensile loading paths. For a variable loading history, a rule of closed hysteresis loops must also be considered [33,34]. Furthermore, special attention has been given to the large nested cycles that do not originate at the envelope of the maximum strain, including a variety of even smaller nested cycles that do not finish at the envelope. Such cycles indicate the modelling capabilities of a material model. The paper is structured as follows. First, the theoretical background for the calculation of the strain–energy density of closed hysteresis loops is given in Section 2. The experimental results are given in Section 3. The parameters of the hysteresis loops for AZ31 are determined in Section 4. In the same section, a comparison between the obtained and predicted fatigue-life results for a variable loading history is given. Section 5 summarises the research outcomes.

## 2. Theoretical Background

### 2.1. Strain–Energy Density of a Closed Hysteresis Loop

Our model for the calculation of the strain–energy density of the closed hysteresis loops for the magnesium alloy is a combination of two different stress–strain functions: a model of the compressive loading path and a model of the tensile loading path. Since the maximum strain amplitudes were smaller than 2%, in our case, the sliding–plasticity mechanism was not activated during the compression phase of the loading cycle. For this reason, only the Ramberg–Osgood equation was applied for the compressive loading path of the stabilised magnesium hysteresis loops:

(3) $$\varepsilon =\frac{\sigma }{E}\;+\;K_{C}\cdot {\left(\frac{\sigma }{E}\right)}^{n_{C}}\;⟶\;\varepsilon \;=\;RO_{C}\left(\sigma \right),$$

where stabilisation represents a half of the fatigue lifetime. On the other hand, the sliding-plasticity mechanism was always activated after the untwining process during the tensile phase of the loading cycle. This is why it was decided on the basis of the literature (Dallmeier et al. [27], Lee et al. [28], and Muhammad et al. [29]) and our own research that a composite model for a tensile loading path of the stabilised Mg-hysteresis loop is used. It is composed of two terms: the Ramberg–Osgood relationship, which describes the untwining mode during the tensile reloading, and the sigmoidal relationship, which describes the transition from the exhaustion of the twinning into the sliding plastification mode:

(4) $$\sigma \;=\;RO_{T}^{-1}\left(\varepsilon \right)\;+\;\frac{B(\Delta \varepsilon_{\max,\mathrm{cyc}})}{1\;+\;\exp\left[-D\cdot \left(\varepsilon \;-\;F(\Delta \varepsilon_{\max,\mathrm{cyc}})\right)\right]}.$$

The term $RO_{T}^{-1}\left(\varepsilon \right)$ in Equation (4) represents an inverse function of the Ramberg–Osgood equation with the material parameters that are characteristic for the tensile loading path of the hysteresis loop:

(5) $$\sigma \;=\;RO_{T}^{-1}\left(\varepsilon \right)\;⟵\;\varepsilon \;=\;RO_{T}\left(\sigma \right)\;=\;\frac{\sigma }{E}\;+\;K_{T}\cdot {\left(\frac{\sigma }{E}\right)}^{n_{T}}.$$

**Figure 2:**
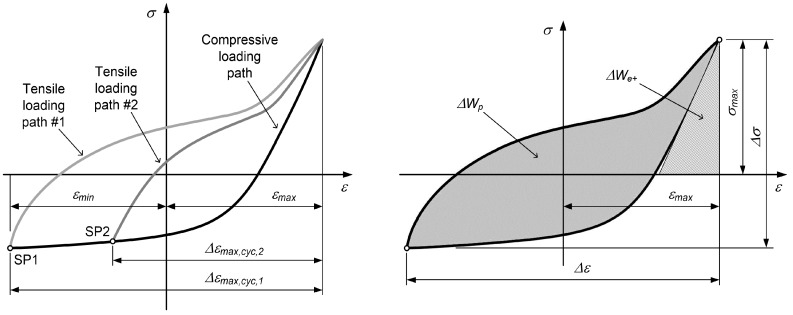
Closed hysteresis loops of magnesium alloy for different strain ranges of low-cycle fatigue tests (left) and energy related to closed hysteresis loops (right).

Since Equation (5) cannot be inverted, the stress σ as a function of strain ε should be calculated numerically. A Newton–Raphson scheme was applied for this purpose, because the function $\sigma \;=\;RO_{T}^{-1}\left(\varepsilon \right)$ is monotonic. Equation (4) is general and is valid for the complete range of strains for the tensile loading paths. The two parameters *B* and *F* depend on the maximum strain $\Delta \varepsilon_{\max,\mathrm{cyc}}$, related to a starting point (SP) of the tensile loading path from the most outside compressive loading path (see the left-hand diagram in Figure 2):

(6) $$B\left(\Delta \varepsilon_{\max,\mathrm{cyc}}\right)\;=\;b_{1}\cdot \left[0.4\;+\;\exp(-b_{2}\cdot \Delta \varepsilon_{\max,\mathrm{cyc}})\right]$$

(7) $$F\left(\Delta \varepsilon_{\max,\mathrm{cyc}}\right)=\left\{\begin{array}{cc} f_{1}\cdot \left(\Delta \varepsilon_{\max,\mathrm{cyc}}\right), & \Delta \varepsilon_{\max,\mathrm{cyc}}\;\;<\;\;f_{2};\\ f_{2}, & \Delta \varepsilon_{\max,\mathrm{cyc}}\;\geq \;f_{2}. \end{array}\right.$$

The parameter *B* describes the increasing and decreasing of the logistic function along the stress σ, while the parameter *F* defines a position of the sigmoidal function along the strain ε. The parameter $f_{2}$ represents a saturation strain for the untwining process within the tensile loading path.

In this manner, the complete model for the stabilised, closed Mg hysteresis loop is composed of only ten material constants: *E*, $K_{C}$, $n_{C}$, $K_{T}$, $n_{T}$, $b_{1}$, $b_{2}$, *D*, $f_{1}$ and $f_{2}$. The parameters can be estimated from the measured closed hysteresis loops at different strain amplitudes with numerical optimisation algorithms. Knowing the material constants, the plastic strain–energy density of a closed hysteresis loop is calculated as

(8) $$\Delta W_{p}\;=\;\int \sigma \left(\varepsilon \right)d\varepsilon$$

where σ is the modelled stress and ε is the given strain in this loop.

### 2.2. Calculating Fatigue-Life with an Energy Approach

According to the selected approach, the energy fatigue-life curve is determined first from the constant strain–amplitude fatigue-life experiments. If the effect of the mean stress is not significant, i.e., if the fatigue loading cycles have approximately equal dynamic coefficients *R*, the energy fatigue-life curve is defined as follows:

(9) $$\Delta W_{p}\cdot N_{f}^{m_{p}}\;=\;C_{p}$$

where $N_{f}$ is the number of loading cycles to failure, $\Delta W_{p}$ is the plastic strain–energy density of the stabilised hysteresis loops, and $m_{p}$ and $C_{p}$ are material constants. If the loading-cycle mean stress needs to be considered, the energy fatigue-life curve is related to the total strain–energy density $\Delta W_{t}$:

(10) $$\Delta W_{t}\cdot N_{f}^{m_{t}}\;=\;C_{t}.$$

The total strain–energy density is the sum of the plastic strain–energy density and the tensile elastic strain energy, as presented in the right-hand diagram in Figure 2 (see also Park et al. [24]):

(11) $$\Delta W_{t}\;=\;\Delta W_{p}\;+\;\Delta W_{e^{+}},$$

(12) $$\Delta W_{e^{+}}\;=\;\frac{\sigma_{\max,\mathrm{cyc}}^{2}}{2\cdot E},$$

where $\sigma_{\max,\mathrm{cyc}}$ is the loading-cycle maximum tensile stress, *E* is the Young’s modulus, and $m_{t}$ and Ct are material constants. To calculate the fatigue life for a variable strain time series, the closed hysteresis loops are first extracted from the time series (see Section 2.3 for details) with the rainflow counting method according to the ASTM E1049 standard [35] or Amzallag et al. [36]. Then, the fatigue life is calculated with the linear damage-accumulation rule (Palmgren–Miner) on the basis of the energy fatigue-life curve from Equation (9) or Equation (10):

(13) $$Dmg\;=\;\sum_{i=1}^{k}\frac{n_{i}}{N_{f}\left(\Delta W_{p/t}^{\left(i\right)}\right)},$$

(14) $$N_{f}\left(\Delta W_{p/t}^{\left(i\right)}\right)\;=\;{\left[\frac{C_{p/t}}{\Delta W_{p/t}^{\left(i\right)}}\right]}^{\frac{1}{m_{p/t}}},$$

where Dmg is the fatigue-life damage and $n_{i}$ is the number of loading cycles at the loading level *k*. In our case, we repeated a block of variable-strain time series until the specimens were broken. In that case, the fatigue life is represented as the number of repeated variable-strain blocks until failure. If the critical fatigue-life damage is defined as $Dmg_{c}$, the number of variable-strain block repetitions to failure rep is equal to:

(15) $$rep\;=\;Dmg_{c}\cdot {\left[\sum_{i=1}^{k}\frac{n_{i}}{N_{f}\left(\Delta W_{p/t}^{\left(i\right)}\right)}\right]}^{-1}.$$

In Equation (15), *k* represents the number of closed hysteresis lops in one variable-strain block.

### 2.3. Modelling the Energy Density of Closed Hysteresis Loops for Variable–Strain Time Series

#### 2.3.1. Extracting Closed Hysteresis Loops of Loading Cycles

**Figure 3:**
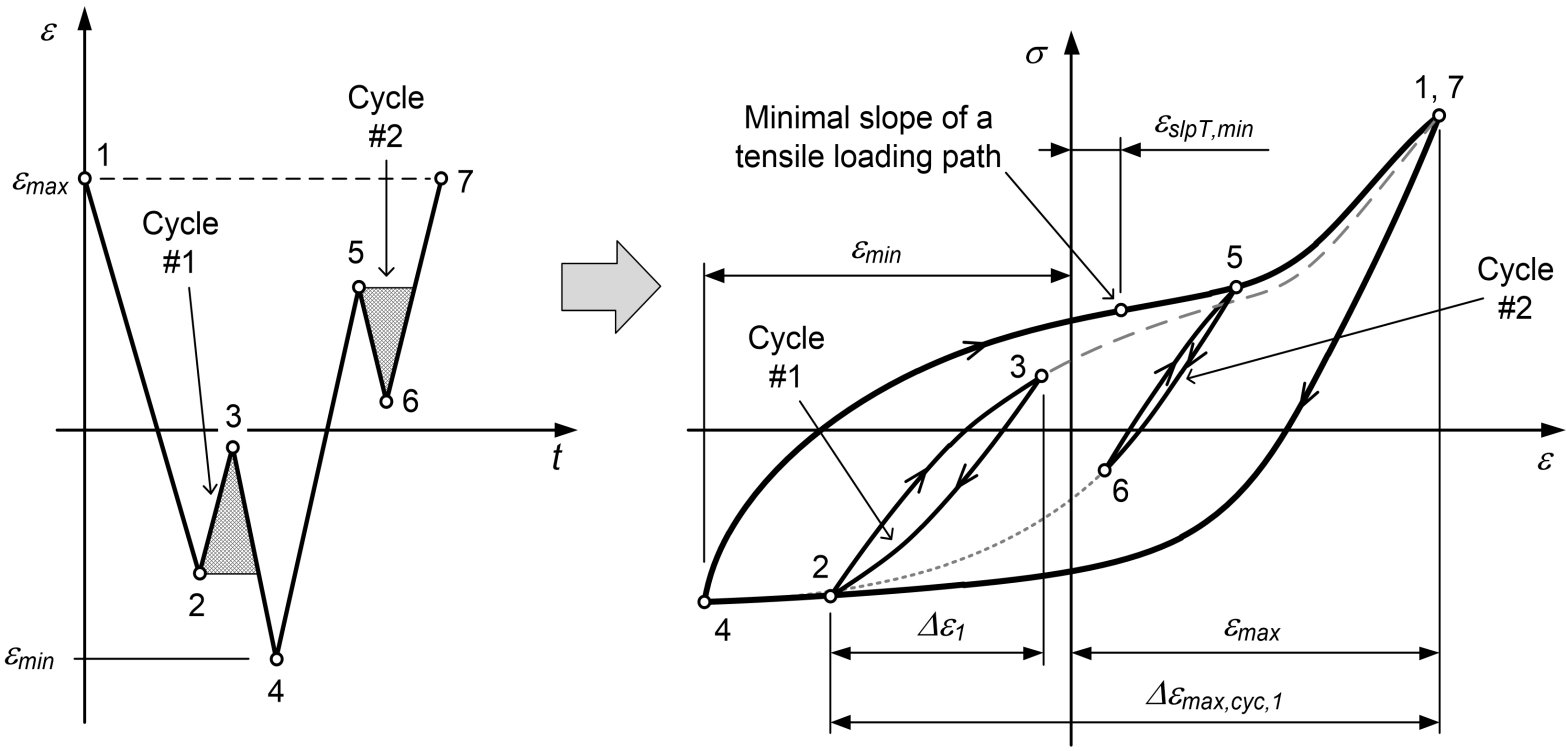
Modelling the loading paths and loading cycles on the basis of the ε(t) time series.

The closed hysteresis loops for the variable ε(t) time series are modelled on the basis of the rainflow counting algorithm according to Amzallag et al. [36]. To avoid the formation of the residuum after the counting, the ε(t) time series is transformed so that it starts and ends with the maximum strain $\varepsilon_{\max}$ (see Figure 3).

With the exception of the largest and the outermost loading cycle, which is defined with Points 1, 4 and 7 in Figure 3, all other loading cycles that form a closed hysteresis loop in the σ–ε diagram must fulfil the condition:

(16) $$\left|\varepsilon_{i-1}-\varepsilon_{i-2}\right|\leq \Delta \varepsilon \;=\;\left|\varepsilon_{i}-\varepsilon_{i-1}\right|\leq \left|\varepsilon_{i+1}-\varepsilon_{i}\right|,$$

where $\varepsilon_{i-2}$ to $\varepsilon_{i+1}$ are four consecutive points in the strain time series. $\varepsilon_{i}$ corresponds to the peak of the standing loading cycle with $\varepsilon_{i}>\varepsilon_{i-1}$ or to the valley of the hanging loading cycle with $\varepsilon_{i}<\varepsilon_{i-1}$ (see Figure 3).

The standing loading cycles are modelled so that first the tensile loading path $\sigma_{T;i-1,i}\left(\varepsilon \right)$ is modelled between the strain points $\varepsilon_{i-1}$ and $\varepsilon_{i}$ with a reference to the preceding compressive loading path that is spanned between the strain points $\varepsilon_{i-2}$ and $\varepsilon_{i-1}$. Then, the reversed compressive loading path $\sigma_{C;i-1,i}\left(\varepsilon \right)$ between the strain points $\varepsilon_{i}$ and $\varepsilon_{i-1}$ is modelled so that the standing loading cycle is completely closed. Similarly, the hanging loading cycles are modelled so that first the compressive loading path $\sigma_{C;i-1,i}\left(\varepsilon \right)$ is modelled between the strain points $\varepsilon_{i-1}$ and $\varepsilon_{i}$ with a reference to the preceding tensile loading path that is spanned between the strain points $\varepsilon_{i-2}$ and $\varepsilon_{i-1}$. Then, the reversed tensile loading path $\sigma_{T;i-1,i}\left(\varepsilon \right)$ between the strain points $\varepsilon_{i}$ and $\varepsilon_{i-1}$ is modelled so that the hanging loading cycle is completely closed. Figure 3 schematically explains this modelling.

If four consecutive strains $\varepsilon_{i-2}$ to $\varepsilon_{i+1}$ in the strain time series are found that contain the closed hysteresis loop according to Equation (16), the loading cycle is completed, its parameters are saved and the strain energy is calculated as presented in Section 2.2. Then, the two points related to the strains $\varepsilon_{i}$ and $\varepsilon_{i-1}$ are removed from the strain time series ε(t) and the current strain point index is returned to the value of i=2. By following this procedure, the closed hysteresis loops are extracted from the strain time series until only three strain points remain. These three points form the outer-most hysteresis loop that is spanned over the complete strain range from $\varepsilon_{\min}$ to $\varepsilon_{\max}$ (see Points 1, 4 and 7 in Figure 3). If the outermost hysteresis increases, then also the minimum and maximum strains $\varepsilon_{\min}$ and $\varepsilon_{\max}$ change.

The tensile and compressive loading paths that are based on Equations (3) and (4) are modelled as described below. To simplify the procedure, the individual elastic–plastic loading paths are shifted fully into the first quadrant of the σ–ε space, as presented in the right-hand diagram of Figure 1 for the plastic loading paths.

#### 2.3.2. Modelling the Tensile Loading Paths

**Figure 4:**
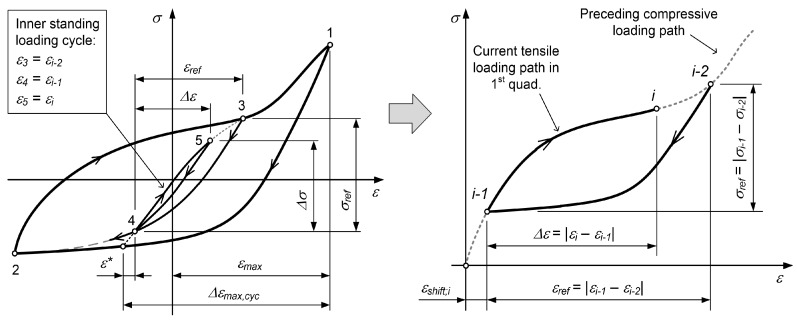
Modelling the tensile loading path that originates from the inner compressive loading path.

**Figure 5:**
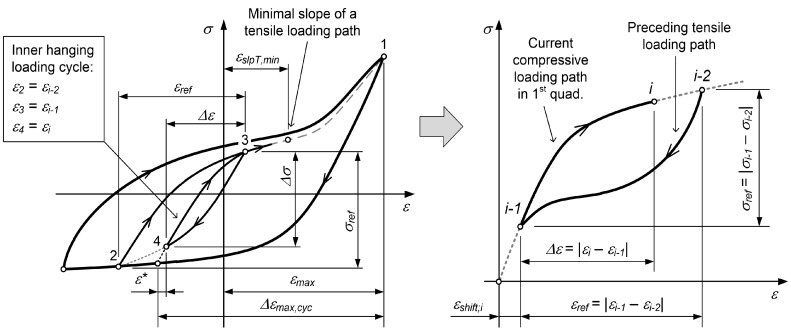
Modelling the compressive loading path that originates from the inner tensile loading path.

The tensile loading path is modelled according to the following procedure:

- If the tensile loading path starts from the value of $\varepsilon_{\min}$, it is the outermost tensile loading path $\sigma_{T;i-1,i}\left(\varepsilon \right)$ with the parameter $\Delta \varepsilon_{\max,\mathrm{cyc}}$ in Equation (4) being equal to $\Delta \varepsilon_{\max,\mathrm{cyc}}\;=\;\left|\varepsilon_{\max}-\varepsilon_{\min}\right|$ (see the σ–ε curve segment 4–7 in Figure 3).
- If the tensile loading path originates from the outermost compressive loading path, but not from $\varepsilon_{\min}$ (as is the case for the segment 2–3 in Figure 3), its tensile loading path $\sigma_{T;i-1,i}\left(\varepsilon \right)$ is modelled with Equation (4) and the parameter $\varepsilon_{\max,\mathrm{cyc}}$ is calculated as follows:
  (17) $$\varepsilon_{\max,\mathrm{cyc}}\;=\;\left|\varepsilon_{\max}-\varepsilon_{i-1}\right|.$$
  According to the case in Figure 3, $\Delta \varepsilon_{\max,\mathrm{cyc}}$ for the first loading cycle is $\Delta \varepsilon_{\max,\mathrm{cyc},1}=\left|\varepsilon_{\max}-\varepsilon_{2}\right|$.
- If the tensile loading path originates from the inner compressive loading path, the corresponding parameter $\Delta \varepsilon_{\max,\mathrm{cyc}}$ is determined first by extending the line with a slope of the elastic modulus *E* from the point $\left(\varepsilon_{i-1},\sigma_{i-1}\right)$ to the outermost compressive loading path (see the left diagram in Figure 4):
  (18) $$\Delta \varepsilon_{\max,\mathrm{cyc}}\;=\;\left|\varepsilon_{\max}-\varepsilon_{i-1}\right|+\left|\varepsilon *\right|$$
  where ε∗ connects the reversal point with the intersection of the tangent to the tensile loading path and the outermost compressive loading path, as shown in Figure 5. The tensile loading path $\sigma_{T;i-1,i}\left(\varepsilon \right)$ of the loading cycle (e.g., the segment 4–5 in Figure 4) is then modelled as a linear combination of the tensile loading path $\sigma_{T}\left(\varepsilon \right)$ from Equation (4) with the corresponding parameter $\Delta \varepsilon_{\max,\mathrm{cyc}}$ from Equation (18) and the compressive loading path $\sigma_{C;i-2,i-1}\left(\varepsilon \right)$ of the previous strain range (the segment 3–4 in Figure 4) with both of the curves modelled in the first quadrant of the σ–ε space:
  (19) $$\sigma_{T;i-1,i}\left(\varepsilon \right)\;=\;w_{T;i}\cdot \left\{RO_{T}^{-1}\left(\varepsilon \right)\;+\;\frac{B(\Delta \varepsilon_{\max,\mathrm{cyc}})}{1+\exp\left[-D\cdot \left(\varepsilon \;-\;F(\Delta \varepsilon_{\max,\mathrm{cyc}})\right)\right]}\right\}\;+\;\left(1\;-\;w_{T,i}\right)\cdot \sigma_{C;i-2,i-1}\left(\varepsilon \right).$$
  The model for the compressive loading path $\sigma_{C;i-2,i-1}\left(\varepsilon \right)$ is described below. The mixing weight $w_{T;i}$ is defined as follows (see also Figure 4):
  (20) $$w_{T;i}\;=\;\frac{\varepsilon_{\mathrm{ref}}}{\Delta \varepsilon_{\max,\mathrm{cyc}}}\;=\;\frac{\left|\varepsilon_{i-1}\;-\;\varepsilon_{i-2}\right|}{\Delta \varepsilon_{\max,\mathrm{cyc}}}.$$
- For the inner loading cycles, part of which is also the tensile loading path, the rule of closed hysteresis loops must hold, as defined by Jayakumar [34]. In our case, this means that a strain shift $\varepsilon_{\mathrm{shift};i}$ must be determined for the inner tensile loading path $\sigma_{T;i-1,i}\left(\varepsilon \right)$ to ensure that its model, which starts at the point $\varepsilon_{i-1}$, reaches exactly the point $\varepsilon_{i-2}$ of the preceding compressive loading path $\sigma_{C;i-2,i-1}\left(\varepsilon \right)$. To calculate the strain shift $\varepsilon_{\mathrm{shift};i}$, the tensile loading path is modelled in the first quadrant of the σ–ε space (see the right-hand diagram in Figure 4 (In general, the quantity $\varepsilon_{\mathrm{shift};i}$ is not equal to the quantity ε∗ in Figure 4)).
- Given that the time series of the previous stresses $\sigma_{i-1}$ to $\sigma_{1}$ corresponding to the strain values $\varepsilon_{i-1}$ to $\varepsilon_{1}$ are known, the highest stress $\sigma_{i}$ in the current tensile loading path that corresponds to the strain $\varepsilon_{i}$ is calculated as follows:
  (21) $$\sigma_{i}\left(\varepsilon_{i}\right)\;=\;\sigma_{i-1}\left(\varepsilon_{i-1}\right)\;+\;\Delta \sigma ,$$
  (22) $$\Delta \sigma \;=\;\left|\sigma_{T;i-1,i}\left(\left|\varepsilon_{i}\;-\;\varepsilon_{i-1}\right|\;+\;\varepsilon_{\mathrm{shift};i}\right)\;-\;\sigma_{T;i-1,i}\left(\varepsilon_{\mathrm{shift};i}\right)\right|.$$

After the tensile loading path $\sigma_{T;i-1,i}\left(\varepsilon \right)$ of the loading cycle and the value of $\sigma_{i}$ are known, either the next compressive loading path $\sigma_{C;i,i+1}\left(\varepsilon \right)$ is determined if it does not close the loading cycle, or the compressive loading path $\sigma_{C;i,i-1}\left(\varepsilon \right)$ is determined that closes the loading cycle.

#### 2.3.3. Modelling the Tensile Loading Paths

The compressive loading path is modelled according to the following procedure:

- If the compressive loading path starts from the value of $\varepsilon_{\max}$, it is the outermost compressive loading path $\sigma_{C;i-1,i}\left(\varepsilon \right)$, which is modelled by Equation (3) (e.g., see the σ–ε curve segment 1–2 in Figure 3).
- If the compressive loading path does not originate from the strain $\varepsilon_{\max}$, regardless of its basic tensile loading path, its model depends on the value of the reference strain $\varepsilon_{\mathrm{ref}}\;=\;\left|\varepsilon_{i-1}\;-\;\varepsilon_{i-2}\right|$. If the reference strain $\varepsilon_{\mathrm{ref}}$ is smaller than the value $\left|\Delta \varepsilon_{\max,\mathrm{cyc}}\;-\;\varepsilon_{\max}\;+\;\varepsilon_{\mathrm{slpT},\min}\right|$ from the preceding tensile loading path (e.g., the σ–ε curve segment 2–3 in Figure 5), the model of the compressive loading path is equal to the model of the preceding tensile loading path, $\sigma_{C;i-1,i}\left(\varepsilon \right)\;=\;\sigma_{T;i-2,i-1}\left(\varepsilon \right)$, but in the opposite direction.
  $\varepsilon_{\mathrm{slpT},\min;i-2,i-1}$ represents the strain that corresponds to the minimum slope of the preceding tensile loading path $\sigma_{T;i-2,i-1}\left(\varepsilon \right)$. If the reference strain $\varepsilon_{\mathrm{ref}}$ is larger than the value $\left|\Delta \varepsilon_{\max,\mathrm{cyc}}\;-\;\varepsilon_{\max}\;+\;\varepsilon_{\mathrm{slpT},\min}\right|$ from the preceding tensile loading path (e.g., the segment 4–5 in Figure 3), the compressive loading path $\sigma_{C;i-1,i}\left(\varepsilon \right)$ is a linear combination of the outermost compressive loading path from Equation (3) (the segment 1–2 in Figure 3 or Figure 5) and the Ramberg–Osgood term from Equation (4):
  (23) $$\sigma_{C;i-1,i}\left(\varepsilon \right)\;=\;w_{C;i}\cdot RO_{C}^{-1}\left(\varepsilon \right)\;+\;\left(1-w_{C;i}\right)\cdot RO_{T;i-2,i-1}^{-1}\left(\varepsilon \right)$$
  The mixing weight $w_{C;i}$ is defined as follows (see also Figure 3 and Figure 5):
  (24) $$w_{C;i}\;=\;\frac{\varepsilon_{\mathrm{ref}}\;-\;\left\{\left|\varepsilon_{\mathrm{slpT},\min;i-2,i-1}\;-\;\varepsilon_{\min}\right|\;-\;\left|\varepsilon_{i-1}-\varepsilon_{\min}\right|\right\}}{\Delta \varepsilon_{\max,\mathrm{cyc}}\;-\;\left\{\left|\varepsilon_{\mathrm{slpT},\min;i-2,i-1}\;-\;\varepsilon_{\min}\right|-\left|\varepsilon_{i-1}\;-\;\varepsilon_{\min}\right|\right\}}.$$
- As with the tensile loading path model, the strain shift $\varepsilon_{\mathrm{shift};i}$ must also be determined for the compressive loading path $\sigma_{C;i-1,i}\left(\varepsilon \right)$ in order for its extension to reach exactly the point $\left(\varepsilon_{i-2},\sigma_{i-2}\right)$ of the preceding tensile loading path $\sigma_{T;i-2,i-1}\left(\varepsilon \right)$ (see Figure 5).
- Given that the time series of the previous stresses $\sigma_{i-1}$ to $\sigma_{1}$ corresponding to the strain values $\varepsilon_{i-1}$ to $\varepsilon_{1}$ are known, the lowest stress $\sigma_{i}$ in the current compressive loading path that corresponds to the strain $\varepsilon_{i}$ is calculated as follows:
  (25) $$\sigma_{i}\left(\varepsilon_{i}\right)\;=\;\sigma_{i-1}\left(\varepsilon_{i-1}\right)\;-\;\Delta \sigma ,$$
  (26) $$\Delta \sigma \;=\;\left|\sigma_{C;i-1,i}\left(\left|\varepsilon_{i}\;-\;\varepsilon_{i-1}\right|\;+\;\varepsilon_{\mathrm{shift};i}\right)\;-\;\sigma_{C;i-1,i}\left(\varepsilon_{\mathrm{shift};i}\right)\right|.$$
  In Equation (25), the compressive loading path must be modelled in the first quadrant of the σ–ε space.

After the compressive loading path $\sigma_{C;i-1,i}\left(\varepsilon \right)$ of the loading cycle and the value of $\sigma_{i}$ are known, either the next tensile loading path for a particular loading cycle $\sigma_{T;i,i+1}\left(\varepsilon \right)$ is determined, if it does not close the loading cycle, or the tensile loading path $\sigma_{T;i,i-1}\left(\varepsilon \right)$ that closes the loading cycle is determined.

#### 2.3.4. Strain–Energy Density Calculation

When all the closed hysteresis loops have been modelled, their plastic strain–energy densities can be calculated according to Equation (8). Using the linear damage-accumulation rule (Palmgren–Miner), the fatigue life can be calculated from Equation (13).

## 3. Experimental Data

**Figure 6:**
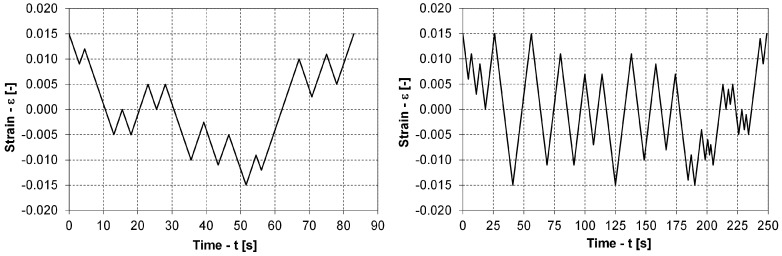
Two variable strain time series for the low-cycle fatigue experiments.

To build a model for predicting the low-cycle fatigue life for variable-strain time series, the following experiments involving the AZ31 magnesium alloy were carried out:

- Static tensile tests were performed up to the rupture of the specimen with an on-line measurement of the engineering stress σ and strain ε during the experiment (four specimens). One specimen was used for metallographic experiments in the zone of maximum strain.
- Strain-controlled fully reversal (R=−1) low-cycle fatigue experiments with constant strain amplitudes $\varepsilon_{a}$ were performed to determine the shape of the closed hysteresis loops at different loading levels and the energy fatigue-life curve: $\varepsilon_{a}=0.25\%$ (four specimens), $\varepsilon_{a}=0.5\%$ (three specimens), $\varepsilon_{a}=0.75\%$ (one specimen), $\varepsilon_{a}=1.00\%$ (three specimens), $\varepsilon_{a}=1.25\%$ (one specimen) and $\varepsilon_{a}=1.5\%$ (one specimen). In each experiment, the number of loading cycles to a break *N* was acquired. One specimen was also used for the metallographic experiments.
- Strain-controlled, fully reversal (R=−1), step-wise, low-cycle fatigue experiments with increasing strain amplitudes $\varepsilon_{a}$ were performed until failure. At each strain amplitude level, 20 loading cycles were completed to stabilise the hysteresis loops. The initial strain amplitude was $\varepsilon_{a}=0.1\%$ and the second strain amplitude level was $\varepsilon_{a}=0.25\%$. After that loading block, the strain–amplitude level was increased by 0.25% in each of the next loading blocks. The step-wise test was terminated after the rupture of the specimen. The purpose of this test was to determine the shape of the closed hysteresis loops and the hardening rule (isotropic or kinematic). Altogether, five step-wise experiments were performed.
- Strain-controlled, variable, low-cycle fatigue experiments for two different strain time histories were performed, as presented in Figure 6. Altogether, two variable-load, low-cycle fatigue experiments were performed between the limit strains of $\varepsilon_{\min}=-1.5\%$ and $\varepsilon_{\max}=1.5\%$ to validate the proposed model for predicting the low-cycle fatigue life of the magnesium alloy AZ31.

**Figure 7:**
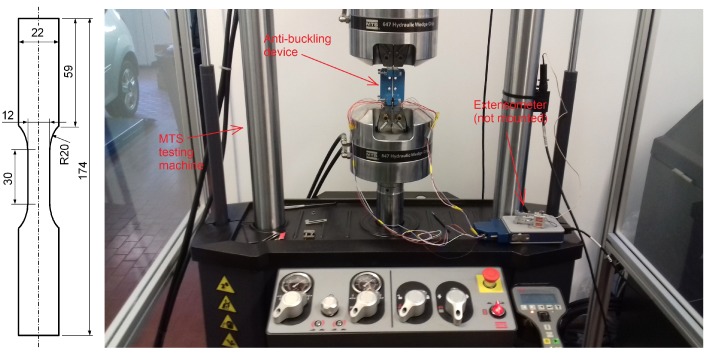
A specimen shape (left) and a low-cycle fatigue experimental arrangement (right).

All specimens were cut from a 2-mm-thick sheet plate of the AZ31 alloy in the “as-received” condition without any additional processing. The dimensions of the sheet plate were 200 mm × 200 mm. The specimens were fine cut with a water jet in the direction of rolling and in the direction perpendicular to the rolling. The specimen surface was not additionally mechanically treated. Their shape and dimensions are presented in Figure 7.

The basic specimen shape, as defined in the ASTM E606 [35], was modified with its dimensions selected in such a manner that: (i) the number of specimens was maximised; (ii) the specimens can be attached to the testing machine; and (iii) an extensometer can be mounted onto the specimens. The tensile and fatigue experiments were carried out on a 100-kN MTS hydraulic testing machine. To measure the strains, an MTS 834.11F-24 extensometer was attached to the middle part of the specimens. The force was measured with a 100-kN tensile-compressive load cell that is integrated into the testing machine. An in-house-developed guiding device was used to prevent any buckling of the specimens [37,38]. The experimental arrangement is presented in Figure 7. The testing frequency for the low-cycle fatigue experiments was varied according to the applied strain–amplitude levels $\varepsilon_{a}$. For the smallest strain amplitudes, the testing frequency was 1 Hz, and, for the highest strain amplitudes, it was 0.1 Hz. The relative experimental errors of the hydraulic test machine were 0.2% and 0.3% for the force and the strain measurements, respectively. The maximum obtained stress was therefore 250±0.5 MPa and the maximum obtained strain was 0.015±0.000045. It was assumed that the experimental error negligibly affected the measurements.

The analysis of the microstructure was performed to corroborate the difference between the deformation mechanisms in AZ31 during the static and dynamic loading, which was the initial motivation for the development of the material model. For the metallographic analysis, the samples were cut in the transversal direction and mounted in Bakelite. The samples were first plane-ground with SiC paper and later fine-ground with a composite disc. For that, a diamond suspension and lubricant were used. Later on, the samples were polished. For polishing, a diamond suspension with a grain size of 1.0 μm was used. The samples were chemically etched with a solution of 99.5% ethanol, distilled water, acetic acid and picric acid. For the microstructure observation, an Olympus BX61 optical microscope (Faculty of Natural Sciences and Engineering, University of Ljubljana, Ljubljana, Slovenia) was used. Scanning electron microscopy (SEM) of the fracture surface was performed on a Jeol JSM 5610 (Faculty of Natural Sciences and Engineering, University of Ljubljana, Ljubljana, Slovenia) under an accelerating voltage of 20 kV after the tensile and fatigue tests.

## 4. Results and Discussion

### 4.1. Experimental Results and Their Modelling

**Figure 8:**
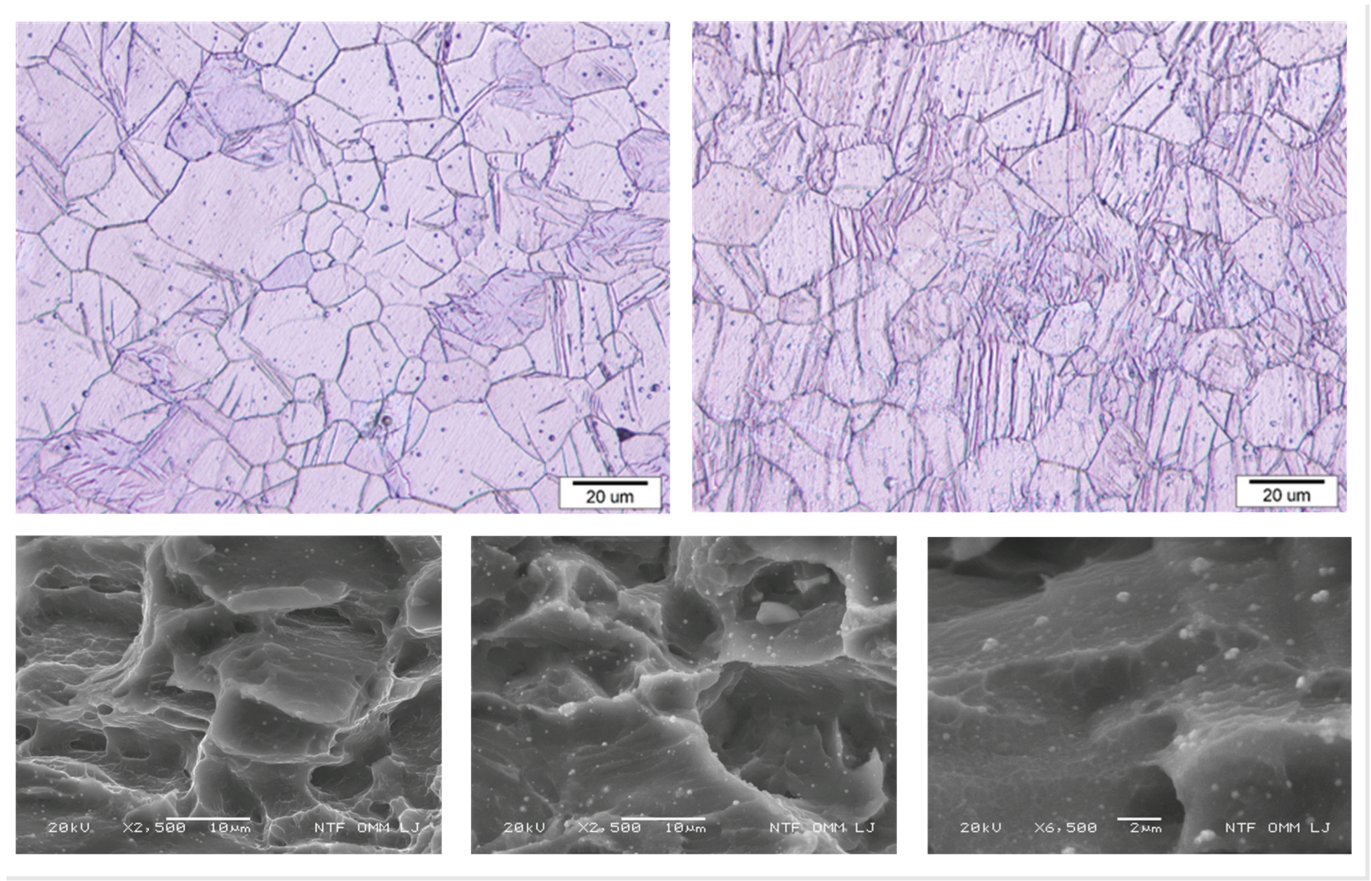
Microstructure of AZ31 specimen by optical microscopy for tensile loading (top left) and low-cycle fatigue loading (top right) and fracture surfaces by SEM for tensile loading (bottom left) and low-cycle fatigue loading (bottom middle and bottom right). Comparison of the microstructure after tensile stress and after low-cycling fatigue clearly shows the difference in the portion of twins remaining in the alloy.

After the static tensile and low-cycle fatigue tests were performed, a portion of each specimen, which was cut out from the most deformed part of the specimen, was crystallographically checked. The pictures of the deformed AZ31 specimens made with the optical microscope and the SEM are presented in Figure 8.

The microstructure after the tensile test (Figure 8, top left) is almost free of twins, since it is known that in magnesium alloys with a c/a ratio of less than 1.732 the twinning occurs under compressive loading perpendicular to the c-axis. The microstructure after the low-cycle fatigue is shown in Figure 8 (top right). Twinning hardly occurs when the sample is under tensile stress. However, when the compressive stress is high enough to activate the twinning, the fraction of twins increases with a higher strain. In tension, most twins disappear; only a small portion of twins remain in the alloy. The final microstructure with a large portion of twins shows that the samples were in the compression state. The fracture surfaces, after both tensile and low-cycle fatigue tests, from the SEM show the characteristics of a trans-granular ductile fracture with micro-void coalescence (Figure 8, bottom left and bottom middle). Additionally, fatigue striations can also be seen in Figure 8 (bottom right).

**Figure 9:**
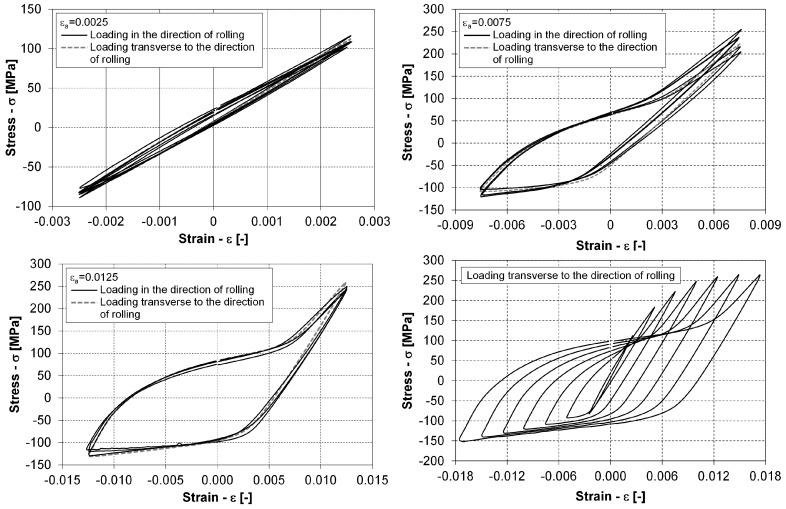
Scatter of stabilised hysteresis loops from different test runs at: $\varepsilon_{a}\;=\;0.25\%$ (top left); $\varepsilon_{a}\;=\;0.75\%$ (top right); and $\varepsilon_{a}\;=\;1.25\%$ (bottom left); and distribution of stabilised hysteresis loops from a step-wise test run (bottom right).

When performing the low-cycle constant-amplitude tests and low-cycle step-wise tests, a considerable scatter in the number of load repetitions until failure was observed. An example of a hysteresis-loop scatter between different tests at an $\varepsilon_{a}\;=\;0.75\%$ strain amplitude is presented in the left-hand diagram of Figure 9. A similar scatter was also observed for the other strain–amplitude levels.

**Figure 10:**
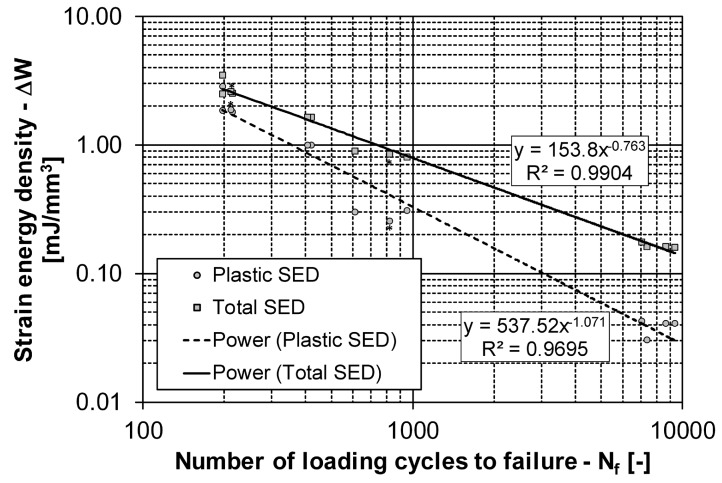
Energy fatigue-life curves: plastic strain–energy density (circles) and total strain–energy density (squares).

There was also a considerable scatter in the measured fatigue lives for the specimens with the same loading level. The range of the fatigue-life scatter can be seen in Figure 10, in which the number of loading cycles to failure is presented for different plastic and total strain–energy densities. We can see in this figure that the scatter is almost half of the order of magnitude along the abscissa axis. This phenomenon was also observed during the step-wise tests that were repeated for five different specimens. Namely, only twice was the fatigue failure at the same loading level, i.e., at $\varepsilon_{a}\;=\;1.25\%$. For the other three specimens, the loading levels at rupture were $\varepsilon_{a}\;=\;0.75\%$, 1.0% and 1.75%. One cause for such fatigue-life scatter is definitely the specimen orientation. For this reason, some specimens were cut from the AZ31 plate at an angle of 90 degrees relative to the rolling direction. These results imply that it is difficult to predict the exact fatigue life for a variable-loaded AZ31 specimen. However, despite the scatter of the fatigue lives, it turned out that the Young’s modulus *E* from the static and the low-cycle tests at small values of the strain was always between E=41 GPa and 45 GPa, with an average value of 43.5 GPa. The latter value was used in further data processing.

**Figure 11:**
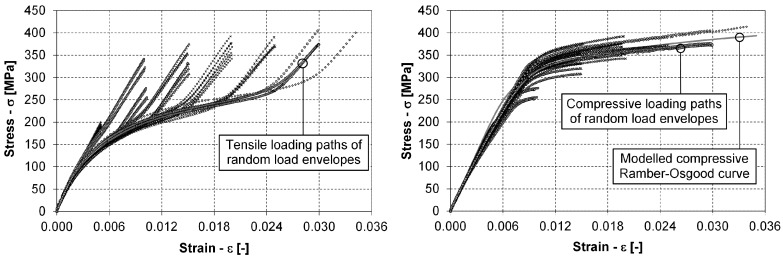
Measured tensile (left) and compressive (right) loading paths from the low-cycle fatigue tests.

In the right-hand diagram of Figure 9, a family of stabilised hysteresis loops from one step-wise experiment is presented. It can be concluded from this diagram that the plastic hardening mechanism is changed if the strain amplitude levels are increased. For small strain amplitudes up to the value of $\varepsilon_{a}\;=\;1.0\%$, the prevailing hardening mechanism is isotropic hardening. Between the strain amplitudes of $\varepsilon_{a}\;=\;1.0\%$ and $\varepsilon_{a}\;=\;1.25\%$, there is a transition from isotropic hardening to kinematic hardening, which prevails at strain amplitudes larger than 1.25%. This conclusion is supported by the right-hand diagram in Figure 11, which shows compressive loading paths from the low-cycle experiments in the first quadrant of the σ–ε space. The changing hardening mechanisms have the following implications for predicting the fatigue life for variable loads:

1. The stabilised hysteresis loops only (up to the outermost hysteresis–loop envelope) should be considered for predicting the number of loading cycles to failure.
2. After a certain strain–amplitude level is reached during the cyclic loading, it is its (the outermost) hysteresis loop that governs the stress–strain behaviour, also for the smaller strain–amplitude loading cycles.
3. The parameters of the Ramberg–Osgood relationship for the outermost compressive loading path that is modelled with Equation (3) should be determined either just for the highest strain amplitude loading level or for all the compressive loading paths from the domain of the kinematic hardening, i.e., $\varepsilon_{a}\;\geq \;1.25\%$ in our case.
4. To build a general model for the tensile loading paths from Equation (4) by considering all the experimental data, the tensile loading paths as presented in the left-hand diagram of Figure 11 must first be extended to the outer-most compressive loading path. Only after this transformation can the parameters of Equation (4) be determined.

**Figure 12:**
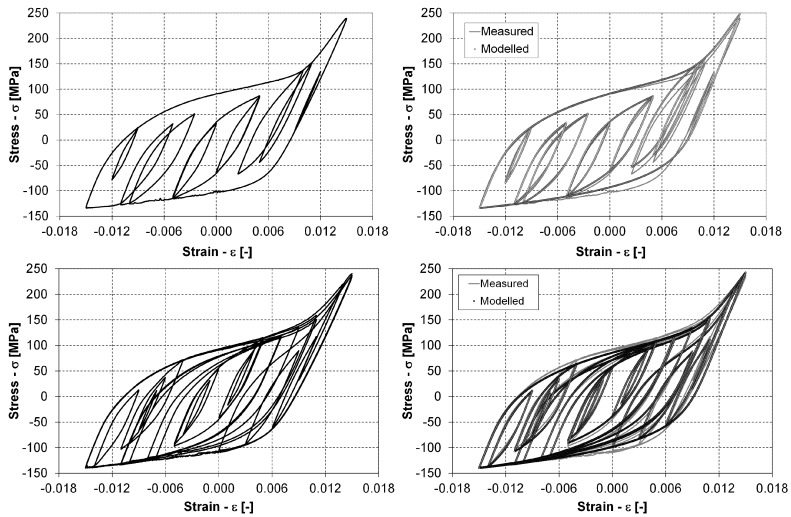
Measured σ–ε response for the simple variable-strain time series (top left); comparison of measured and modelled σ–ε responses for the simple variable-strain time series (top right); measured σ–ε response for the complex variable-strain time series (bottom left); and comparison of measured and modelled σ–ε responses for the complex variable-strain time series (bottom right).

Finally, two variable-strain time-series experiments were performed. The stabilised σ–ε response for the complete period of the simpler variable test is presented at the top of Figure 12. This response corresponds to the ε(t) time series from the left-hand diagram of Figure 6, which is derived from one of the time series from [27]. The stabilised σ–ε response for the complete period of the second variable signal in Figure 6 is presented at the bottom of Figure 12.

We can see from Figure 12 that the tensile and compressive loading paths form an envelope for all the other hysteresis loops that are present in the two variable signals. We included the outermost tensile and compressive loading paths from the two variable tests into Figure 11 to compare them with the constant strain–amplitude experiments. It can be seen from both diagrams in Figure 11 that the loading paths from the variable test agree well with all the other low-cycle fatigue data. It took 29 repetitions of the period for the first variable load until the fatigue failure, but only 13 repetitions until failure for the second variable load. In both cases, the σ–ε response for the complete loading period was stabilised after three loading cycles. For this reason, the stabilised σ–ε response at the half repetitions of the variable signals was considered for the data analysis, i.e., the 15th loading period for the first variable load and the 7th loading period for the second variable load. The model for the compressive loading paths according to Equation (3) was built first. The parameters $n_{C}$ and $K_{C}$ were estimated with a real-valued genetic algorithm (RVGA) that was applied before for modelling different fatigue-life curves and their scatter. For details on the theoretical background and the selected parameters that govern the optimisation procedure, see the works of Klemenc and Fajdiga [39] and Klemenc [40]. The objective function was the sum of the squared distances between the measured and the modelled data points:

(27) $$SSQD\;=\;\sum_{j=1}^{k_{C}}{\left[\sigma_{j}-RO_{C}^{-1}\left(\varepsilon_{j}\right)\right]}^{2}.$$

The training database consisted of $k_{C}\;=\;704$ data points $\left(\varepsilon_{j},\sigma_{j}\right)$ that belong to all the measured compressive loading paths of the constant amplitude tests, the step-wise tests and the compressive envelopes of the two variable σ–ε responses for $\varepsilon_{a}\;\geq \;1.25\%$. Ten RVGA runs with variable initial conditions and 10,000 iterations were made to reach the final estimates of the $n_{C}$ and $K_{C}$ parameters for the value of the Young’s modulus E=43,500 MPa (see Table 1). The model of the compressive loading path is presented in the right-hand diagram of Figure 11. It can be seen in this figure that the model represents the data well and is a good trade-off between the compressive loading paths from different kinds of low-cycle fatigue experiments.

After the model for the compressive loading paths was built, the tensile loading paths from the left-hand diagram in Figure 11 were first scaled so that their highest points coincide with the modelled compressive loading path. Then, the scaled tensile-loading paths from all the experiments (the constant amplitude tests, the step-wise tests and the compressive envelopes of the two variable σ–ε responses) were joined into a training database with $k_{T}\;=\;3632$ data points $\left(\varepsilon_{j},\sigma_{j}\right)$. To each data point, a weight was added to improve the parameter-estimation procedure, since the density of the data points is much smaller at the end of the loading paths. The data-point weight $w_{\mathrm{DP};j}$ depended on the data point’s stress magnitude:

(28) $$w_{\mathrm{DP};j}\;=\;1.0\;+\;3.0\cdot \frac{\sigma_{j}\;-\;\sigma_{\min}}{\sigma_{\max}\;-\;\sigma_{\min}};\;\;j\;=\;1,\ldots ,k_{T}.$$

With such a modification, the contribution of the data points at different stress levels is approximately equalised, because the point density along the stress axis varies significantly.

Finally, the parameters $K_{T}$, $n_{T}$, $b_{1}$, $b_{2}$, *D*, $f_{1}$ and $f_{2}$ of the tensile loading paths were estimated with the same version of the RVGA as before. The objective function was a weighted sum of the squared distances between the measured and the modelled data points, but for the tensile loading path in this case the following was applied:

(29) $$SSQD\;=\;\sum_{j=1}^{k_{C}}w_{\mathrm{DP};j}\cdot {\left[\sigma_{j}\;-\;\left\{RO_{T}^{-1}\left(\varepsilon_{j}\right)\;+\;\frac{B(\Delta \varepsilon_{\max,\mathrm{cyc}})}{1+\exp\left[-D\cdot \left(\varepsilon_{j}\;-\;F\left(\Delta \varepsilon_{\max,\mathrm{cyc}}\right)\right)\right]}\right\}\right]}^{2}.$$

Ten RVGA runs with variable initial conditions and 10,000 iterations were made to reach the final estimates of the seven parameters $K_{T}$, $n_{T}$, $b_{1}$, $b_{2}$, *D*, $f_{1}$ and $f_{2}$ (see the last column in Table 1). Again, the model represents the data well and is a good trade-off between the tensile loading paths from different types of low-cycle fatigue experiments. The tensile loading paths are not presented in the article in order to avoid data overload in the figures. The value of the parameter $f_{2}$ in Table 1 is put into parentheses because it is less reliable than the other parameters. The reason for this is that there was no well-expressed untwining saturation in the tensile loading paths below the maximum strain amplitude of $\varepsilon_{a}\;=\;2.0\%$.

### 4.2. Prediction of Fatigue Life Using the Strain–Energy Density

From the 13 fatigue-life experiments with a constant amplitude strain, the energy fatigue–life curves were estimated. As described in Section 2.2, the plastic strain–energy density $\Delta W_{p}$ and total strain–energy density $\Delta W_{t}$ were calculated from the stabilised hysteresis loops at the half fatigue life. In Figure 10, the $\left(\Delta W_{p/t},N_{f}\right)$ points are presented together with the corresponding energy fatigue–life curves. The material-dependent parameters of the two energy fatigue-life curves are as follows:

- plastic strain–energy density $\Delta W_{p}$: $C_{p}\;=\;537.52$, $m_{p}\;=\;1.0705$; and
- total strain–energy density curve $\Delta W_{t}$: $C_{t}\;=\;153.80$, $m_{t}\;=\;0.7627$.

These energy curves were applied to predict the fatigue-life damage for the two variable-strain time series. To predict the fatigue life, the closed hysteresis loops were first extracted from the ε(t) history, which was followed by modelling their tensile and compressive loading paths, as described in Section 2.3. There are $k_{\mathrm{cyc}}\;=\;10$ closed hysteresis loops in the first variable signal and $k_{\mathrm{cyc}}\;=\;21$ hysteresis loops in the second variable signal. The modelled hysteresis loops for both the variable signals are presented in the right-hand diagrams of Figure 12. We can conclude from the results in Figure 12 that the agreement between the measured and the modelled σ–ε responses is very good if we consider the amount of scatter related to the fatigue-life experiments. The only significant deviation between the measured and modelled responses is in the lower-right-hand corner of the two diagrams, where the compressive loading path changes its slope. Even in this area, the deviation between the measured and modelled responses is less than 10% along the stress axis. The quality of the agreement is confirmed by the data in Table 2 and Table 3. If we compare the maximum stresses $\sigma_{\max}$ of the closed hysteresis loops, we can see that the deviation between the measured and modelled data is always less than 10% for the first variable strain history, and that only four out of 21 hysteresis loops have the deviation of the maximum stress larger than 10%. However, these are the smallest loading cycles that do not influence the fatigue life significantly.

After the hysteresis loops were extracted from the measured and modelled σ–ε responses, the strain–energy densities $\Delta W_{p}$ and $\Delta W_{t}$ were calculated for each hysteresis loop. In Table 2, the calculated plastic and total strain–energy densities are listed for the measured and modelled hysteresis loops of the first variable-strain history. In Table 3, the same data are listed for the second variable-strain history. The comparison between the measured and the modelled values are in good agreement, apart from a few smallest cycles which negligibly contribute to the total damage.

The energy fatigue-life curves in Figure 10 were applied to calculate the damage $Dmg_{i}$ on the basis of the data in Table 2 and Table 3. The fatigue-life damage that is caused by one repetition of each variable-strain history is equal to:

(30) $$Dmg\;=\;\sum_{i=1}^{k_{\mathrm{cyc}}}Dmg_{i}$$

If the critical fatigue damage $Dmg_{c}$ is equal to 1.0, the predicted number of variable-signal repetitions until fatigue failure is (see also Section 2.2):

(31) $$rep\;=\;\frac{Dmg_{c}}{Dmg}\;=\;\frac{1.0}{Dmg}.$$

During the low-cycle fatigue testing, the first (simpler) variable-strain history was repeated 29 times until the failure of the specimen. The predicted fatigue life on the basis of the plastic strain–energy density $\Delta W_{p}$ was 60 repetitions until failure for the measured and modelled data. On the other hand, the predicted fatigue life on the basis of the total strain–energy density $\Delta W_{t}$ was 74 repetitions for the measured data and 73 repetitions for the modelled data. The second variable-strain history was repeated 13 times until failure. The predicted fatigue life on the basis of the plastic strain–energy density was 15 (measured data) and 16 (modelled data) loading cycles. If the total strain–energy density was considered, the predictions were 18 (measured data) and 20 (modelled data) loading cycles. We can see that the predictions were always non-conservative. Regardless of the method, the fatigue-life predictions from the measured and modelled data differed by no more than two repetitions of the variable-strain history. This is another confirmation that the presented model of the AZ31 hysteresis loops is very good. From the presented results, it can be concluded that in the case of the low-cycle fatigue regime the AZ31 magnesium alloy does not show a significant mean-stress effect, because for both variable-strain histories consistently better fatigue-life predictions were made on the basis of the plastic strain–energy density $\Delta W_{p}$. Since the scatter of the energy fatigue-life curve along the number of loading cycles to failure *N* is considerable (see Figure 10), the prediction is in its scatter range, even for the first variable signal, despite the fact that the predicted fatigue life was twice the measured fatigue life. This means that the energy-based approach combined with the presented model of the hysteresis loops is appropriate for predicting the fatigue life of the AZ31 magnesium alloy.

The prediction model has been applied to AZ31 magnesium alloy during this study. However, the methodology is applicable also to other materials that exhibit a similar asymmetric stress–strain response. The material parameters of the model have to be determined from the LCF tests for the material under investigation and then the stress–strain response and the fatigue damage can be predicted.

## 5. Conclusions

In the article, a method for predicting the fatigue life of magnesium alloys is presented, based on the energy approach. With the introduction of an analytical model for describing the tensile and compressive loading paths of the closed hysteresis loops, it is possible to predict the fatigue life for an arbitrary strain–loading history on the basis of the plastic or total strain–energy density. The research outcomes that are presented in the article can be summarised as follows:

- A new model for tensile and compressive elastic–plastic hysteresis loops is presented, which enables the modelling of an arbitrary hysteresis loop in the low-cycle fatigue domain with only 10 parameters.
- Static, dynamic and metallographic experiments were carried out to determine the elastic–plastic response of the AZ31 alloy and to estimate the influence of the loading direction on the microstructure of thin AZ31 sheets.
- The presented hysteresis-loop model was validated against experimental fatigue-life data that emerge from different tests: constant strain–amplitude testing, step-wise variation of the strain–amplitude levels and the arbitrary variation of strain amplitudes in a variable signal.
- An energy fatigue-life curve was determined on the basis of the experimental data that were applied for predicting the fatigue life in the low-cycle fatigue domain.
- It was proven that the fatigue lives that are predicted on the basis of the modelled hysteresis loops are equivalent to the fatigue lives that are predicted on the basis of the measured stress–strain response.
- It was shown that better fatigue-life predictions were obtained on the basis of the plastic strain–energy densities than the total strain–energy densities.

## Figures and Tables

**Table 1:** Parameters of the tensile and compressive loading path models.

Parameter Name	Compressive Loading Path		Tensile Loading Path	
Elastic Modulus	*E* [MPa]	43,500	*E* [MPa]	43,500
Hardening Exponent	$n_{C}$ [-]	9.5974	$n_{T}$ [-]	4.2376
Yield-stress-dependent Parameter	$K_{C}$ [-]	$1.1561\times 10^{18}$	$K_{T}$ [-]	$4.8327\times 10^{7}$
Parameters of Sigmoid Function			$b_{1}$ [-]	193.88
			$b_{2}$ [-]	28.395
			*D* [-]	523.29
			$f_{1}$ [-]	0.95959
			$f_{2}$ [-]	(0.38102)

**Table 2:** Comparison of measured and modelled strain energy densities–variable strain history #1.

Strain Cycles		Measured Data				Modelled Data				Relative Error			
Cycle	$\varepsilon_{a}$	$\sigma_{\max}$	$\Delta W_{p}$	$\Delta W_{e^{+}}$	$\Delta W_{t}$	$\sigma_{\max}$	$\Delta W_{p}$	$\Delta W_{e^{+}}$	$\Delta W_{t}$	$\sigma_{\max}$	$\Delta W_{p}$	$\Delta W_{e^{+}}$	$\Delta W_{t}$
[i]	[-]	[MPa]	[$\frac{\mathrm{mJ}}{{\mathrm{mm}}^{3}}$]	[$\frac{\mathrm{mJ}}{{\mathrm{mm}}^{3}}$]	[$\frac{\mathrm{mJ}}{{\mathrm{mm}}^{3}}$]	[MPa]	[$\frac{\mathrm{mJ}}{{\mathrm{mm}}^{3}}$]	[$\frac{\mathrm{mJ}}{{\mathrm{mm}}^{3}}$]	[$\frac{\mathrm{mJ}}{{\mathrm{mm}}^{3}}$]	[-]	[-]	[-]	[-]
1	0.0150	239.3	4.101	0.658	4.760	247.8	4.055	0.706	4.761	0.035	0.011	−0.072	0.000
2	0.0015	134.5	0.023	0.208	0.231	131.7	0.001	0.196	0.196	−0.020	0.956	0.059	0.152
3	0.0025	32.6	0.139	0.012	0.151	35.5	0.146	0.014	0.160	0.090	−0.047	−0.188	−0.059
4	0.0050	86.9	0.685	0.087	0.771	86.2	0.657	0.085	0.742	−0.009	0.041	0.017	0.038
5	0.0025	86.0	0.128	0.085	0.213	86.1	0.145	0.085	0.230	0.001	−0.135	−0.003	−0.082
6	0.0038	51.6	0.352	0.031	0.383	51.7	0.375	0.031	0.406	0.002	−0.066	−0.005	−0.061
7	0.0030	32.1	0.211	0.012	0.222	33.8	0.229	0.013	0.243	0.053	−0.090	−0.109	−0.091
8	0.0015	23.2	0.039	0.006	0.046	25.5	0.032	0.007	0.039	0.099	0.194	−0.207	0.139
9	0.0037	136.8	0.311	0.215	0.526	148.4	0.315	0.253	0.568	0.085	−0.015	−0.177	−0.081
10	0.0030	151.5	0.122	0.264	0.386	162.0	0.153	0.302	0.455	0.069	−0.259	−0.144	−0.180

**Table 3:** Comparison of measured and modelled strain energy densities–variable strain history #2.

Strain Cycles		Measured Data				Modelled Data				Relative Error			
Cycle	$\varepsilon_{a}$	$\sigma_{\max}$	$\Delta W_{p}$	$\Delta W_{e^{+}}$	$\Delta W_{t}$	$\sigma_{\max}$	$\Delta W_{p}$	$\Delta W_{e^{+}}$	$\Delta W_{t}$	$\sigma_{\max}$	$\Delta W_{p}$	$\Delta W_{e^{+}}$	$\Delta W_{t}$
[i]	[-]	[MPa]	[$\frac{\mathrm{mJ}}{{\mathrm{mm}}^{3}}$]	[$\frac{\mathrm{mJ}}{{\mathrm{mm}}^{3}}$]	[$\frac{\mathrm{mJ}}{{\mathrm{mm}}^{3}}$]	[MPa]	[$\frac{\mathrm{mJ}}{{\mathrm{mm}}^{3}}$]	[$\frac{\mathrm{mJ}}{{\mathrm{mm}}^{3}}$]	[$\frac{\mathrm{mJ}}{{\mathrm{mm}}^{3}}$]	[-]	[-]	[-]	[-]
1	0.0150	237.7	4.175	0.649	4.824	242.3	4.063	0.675	4.738	0.019	0.027	−0.039	0.018
2	0.0025	117.5	0.069	0.159	0.228	111.1	0.122	0.142	0.264	−0.055	−0.771	0.106	−0.160
3	0.0030	89.6	0.188	0.092	0.280	86.0	0.217	0.085	0.302	−0.041	−0.155	0.080	−0.077
4	0.0075	237.2	1.160	0.647	1.807	242.3	1.122	0.675	1.797	0.021	0.033	−0.043	0.006
5	0.0150	240.4	4.285	0.664	4.950	241.8	4.054	0.672	4.726	0.005	0.054	−0.011	0.045
6	0.0110	150.9	2.751	0.262	3.013	151.4	2.532	0.264	2.795	0.004	0.080	−0.008	0.072
7	0.0090	117.7	1.969	0.159	2.128	110.9	1.833	0.141	1.975	−0.058	0.069	0.113	0.072
8	0.0070	118.8	1.298	0.162	1.460	110.8	1.201	0.141	1.342	−0.067	0.075	0.129	0.081
9	0.0130	158.7	3.521	0.289	3.810	156.5	3.321	0.281	3.602	−0.014	0.057	0.027	0.055
10	0.0095	135.0	2.174	0.209	2.383	134.2	2.089	0.207	2.296	-0.006	0.039	0.012	0.037
11	0.0075	118.8	1.446	0.162	1.608	120.1	1.415	0.166	1.581	0.010	0.021	−0.021	0.017
12	0.0025	12.6	0.145	0.002	0.147	10.8	0.142	0.001	0.144	−0.139	0.020	0.259	0.023
13	0.0035	72.0	0.296	0.060	0.355	63.4	0.325	0.046	0.371	−0.119	−0.098	0.225	−0.044
14	0.0020	41.9	0.084	0.016	0.100	34.3	0.080	0.013	0.094	−0.078	0.042	0.149	0.059
15	0.0010	11.6	0.018	0.002	0.020	6.0	0.007	0.000	0.007	−0.480	0.633	0.729	0.641
16	0.0025	114.4	0.122	0.150	0.273	108.0	0.131	0.134	0.264	−0.056	−0.068	0.109	0.030
17	0.0015	92.3	0.038	0.098	0.136	90.8	0.034	0.095	0.129	−0.017	0.092	0.033	0.049
18	0.0050	113.9	0.686	0.149	0.836	108.1	0.646	0.134	0.780	−0.051	0.060	0.100	0.067
19	0.0025	56.0	0.146	0.036	0.182	56.6	0.145	0.037	0.182	0.009	0.005	−0.019	0.000
20	0.0015	31.1	0.037	0.011	0.048	36.4	0.034	0.015	0.049	0.169	0.081	−0.366	−0.023
21	0.0025	218.9	0.050	0.551	0.601	219.2	0.001	0.481	0.481	0.002	0.980	0.126	0.199

## Abbreviations

The following abbreviations are used in this manuscript:

$b_{1}$	First parameter of the scaling term in the sigmoid function
$b_{2}$	Second parameter of the scaling term in the sigmoid function
$f_{1}$	First parameter of the shifting term in the sigmoid function
$f_{2}$	Second parameter of the shifting term in the sigmoid function
*k*	Number of levels or data points
*n*	Hardening exponent in the Ramberg–Osgood equation
rep	Number of repetitions
*w*	Weighting factor
*A*	Scaling parameter for the exponential function
*B*	Scaling parameter for the strain in the exponential function, scaling term in the sigmoid function
*D*	Scaling parameter for the strain in the sigmoid function
Dmg	Fatigue damage
Dmgc	Critical fatigue damage
*E*	Young’s modulus
*F*	Shifting term in the sigmoid function
*K*	Parameter of the Ramberg–Osgood equation related to the yield strength
$N_{f}$	Number of loading cycles to failure
*R*	Load ratio
SSQD	Sum of squared distances
*e*	Strain
e∗	Extension of the partial tensile loading path to the outer-most compressive loading path along the strain axis
$e_{\mathrm{shift}}$	Strain shift for the tensile/compressive loading path to ensure closing of the hysteresis loop
*s*	Stress
*D*	Increment or interval width
$D_{W}$	Strain–energy density related to one hysteresis loop
$D_{e}$	Strain range
$D_{\mathrm{emax},\mathrm{cyc}}$	Strain range for the complete tensile loading path
$D_{s}$	Stress range
*a*	Index related to the amplitude quantity
e+	Index related to the elastic strain–energy density
i,j	Running indices
max	Maximum value of the quantity
min	Minimum value of the quantity
p	Index related to the plastic strain–energy density
t	Index related to the total strain–energy density
C	Index related to the compressive loading path
DP	Index related to the data point
T	Index related to the tensile loading path
